# Where Do Livestock Guardian Dogs Go? Movement Patterns of Free-Ranging Maremma Sheepdogs

**DOI:** 10.1371/journal.pone.0111444

**Published:** 2014-10-29

**Authors:** Linda van Bommel, Chris N. Johnson

**Affiliations:** 1 School of Zoology, University of Tasmania, Hobart, Australia; 2 Fenner School of Environment and Society, Australian National University, Canberra, Australia; Institut Pluridisciplinaire Hubert Curien, France

## Abstract

In many parts of the world, livestock guardian dogs (LGDs) are a relatively new and increasingly popular method for controlling the impact of wild predators on livestock. On large grazing properties in Australia, LGDs are often allowed to range freely over large areas, with minimal supervision by their owners. How they behave in this situation is mostly unknown. We fitted free-ranging Maremma sheepdogs with GPS tracking collars on three properties in Victoria, Australia; on two properties, four sheep were also fitted with GPS collars. We investigated how much time the Maremmas spent with their livestock, how far they moved outside the ranges of their stock, and tested whether they use their ranges sequentially, which is an effective way of maintaining a presence over a large area. The 95% kernel isopleth of the Maremmas ranged between 31 and 1161 ha, the 50% kernel isopleth ranged between 4 and 252 ha. Maremmas spent on average 90% of their time in sheep paddocks. Movements away from sheep occurred mostly at night, and were characterised by high-speed travel on relatively straight paths, similar to the change in activity at the edge of their range. Maremmas used different parts of their range sequentially, similar to sheep, and had a distinct early morning and late afternoon peak in activity. Our results show that while free-ranging LGDs spend the majority of their time with livestock, movements away from stock do occur. These movements could be important in allowing the dogs to maintain large territories, and could increase the effectiveness of livestock protection. Allowing LGDs to range freely can therefore be a useful management decision, but property size has to be large enough to accommodate the large areas that the dogs use.

## Introduction

Livestock Guardian Dogs (LGDs, *Canis familiaris*) are usually dogs of large breeds that are kept with livestock to protect them from predators. Most LGD breeds originated in Europe and Asia, where they have been used for centuries to protect livestock from predators and thieves. They are raised with stock from an early age, and as a result view livestock as their social companions and protect them from threats [1]–[3]. Experimental and anecdotal evidence shows that these dogs can be effective in protecting a range of livestock species from several types of predators (older studies reviewed in [4], see also [5]–[9]). In Australia, as in many other parts of the world outside their countries of origin, LGDs are a relatively new, and increasingly popular method to reduce predation on livestock [10], [11]. LGDs can be fence-trained so they will remain in the paddock in which their livestock are confined, but they can also be allowed to cross stock fences to roam more freely [11], [12]. This free-ranging system is often used on large grazing properties in Australia, where the main threat is posed by feral dogs, domestic dogs, dingoes and their hybrids (*Canis familiaris*, and *Canis dingo,* are hereafter referred to as wild dogs). In these situations, LGDs can potentially roam over large areas. LGDs might be visited by their owners intermittently, sometimes less than once a week [10], and the owners are mostly unaware of their LGDs' movements. Free-ranging could have a significant behavioural function for LGDs, such as maintaining usage of a territory around the livestock from which wild predators are excluded. On the other hand, roaming by LGDs could potentially leave livestock vulnerable, and cause other problems, such as creating traffic hazards or having negative impacts on wildlife [6], [12], [13].

In this study, GPS collars were used to study the movements and behaviour of free-ranging Maremma sheepdogs on three properties in Victoria, Australia. On two properties sheep *Ovis aries* were also fitted with GPS collars. We had several specific aims. First, we wanted to know the size of the Maremmas' range to determine how much space these dogs used. We also wanted to know if the Maremmas' behaviour differed with regard to proximity to the core of their range. We measured their behaviour through their speed of movement (m/h) and the straightness versus tortuosity of their movement path. We expected to find relatively low-speed movement in a tortuous path at the core of the range, and high movement speed in a relatively straight line at the edge of their range, reflecting behaviour such as boundary patrolling or seeing off predators at the edge of the range versus resting or attending livestock at the core. Second, we wanted to determine the proportion of time the Maremmas spent with livestock, at what times of the day they were most likely to leave them, and if their behaviour changed when they left the livestock. We expected higher movement speeds in a relatively straight line away from stock versus low movement speeds in a more tortuous path close to stock, similar to the change in behaviour at the edge of the range versus the core. Third, we tested if Maremmas used their range sequentially. Sequential use of a territory indicates that a different part of the range is patrolled each day, and can provide an effective way for an animal to establish a presence over a large area. We also determined their 24-hour activity pattern in order to compare this to the activity patterns of the stock and predators.

## Materials and Methods

### Ethics statement

All research was carried out in compliance with the Australian Code for the Care and Use of Animals for Scientific Purposes, 7^th^ edition. Ethics approval was obtained from the Animal Ethics Committee of the University of Tasmania (approval number: A0012323).

### Study sites

**Figure 1:**
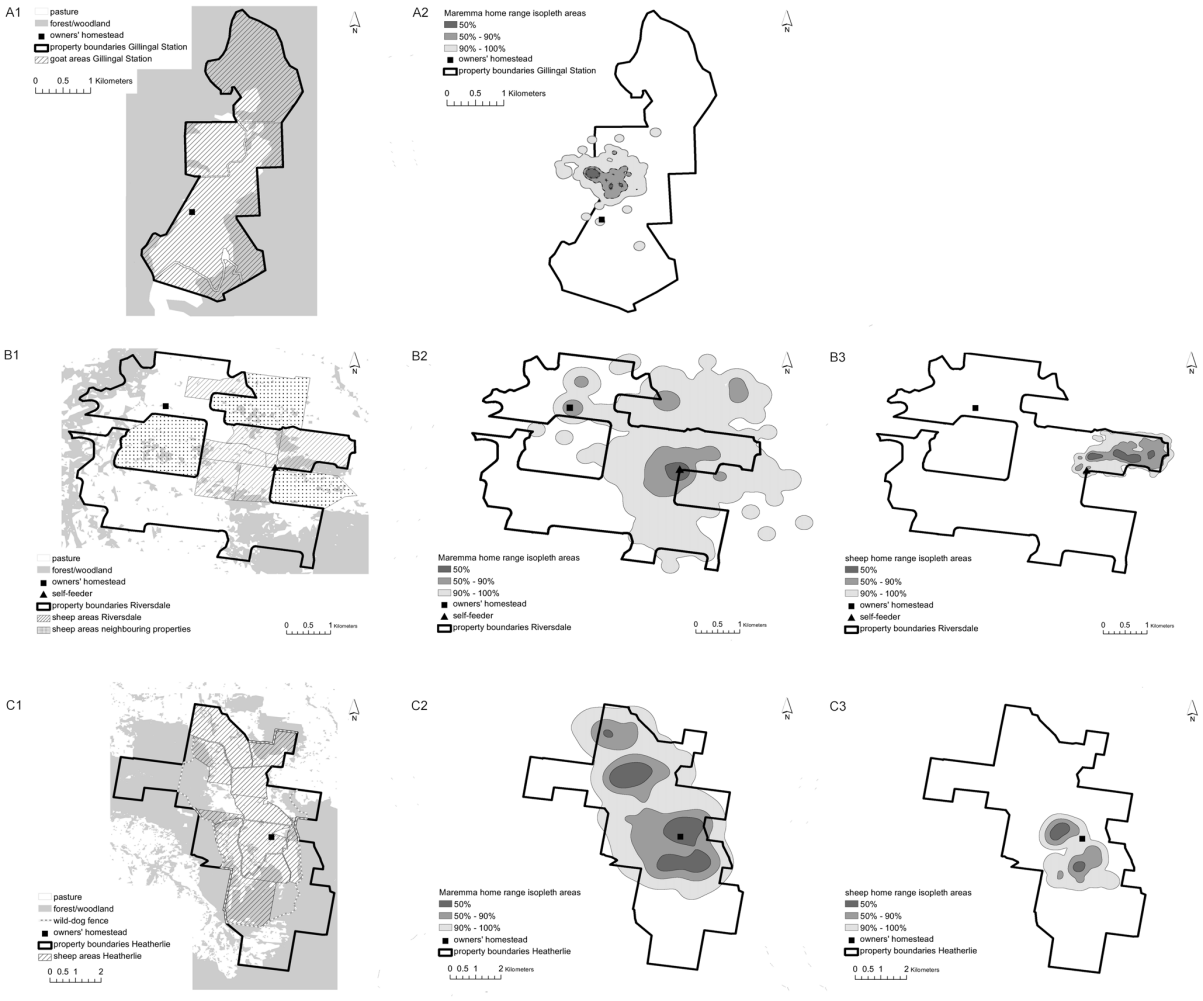
The three research properties used in this study. A. Gillingal Station, B. Riversdale, C. Heatherlie. Image 1 of each property shows the vegetation on the sites, and the areas where the sheep or goats were grazed. In addition, for Heatherlie, the position of the electrified wild-dog fence is indicated. Image 2 of each property shows a representative example of a Maremma home range, depicting the 50%, 50%–90% and 90%–100% kernel isopleth areas. In this figure, tracking data was used from a Maremma in 2010 on Riversdale and in 2011 on Heatherlie, to coincide with sheep home ranges depicted in image 3. Image 3 of Riversdale and Heatherlie shows a representative example of a sheep home range, depicting the 50%, 50%–90% and 90%–100% kernel isopleth areas.

We studied Maremma sheepdogs on three properties in Victoria, Australia. Gillingal Station was situated in eastern Victoria, and Riversdale and Heatherlie were situated in northeast Victoria, approximately 15 km apart. Details of these properties and management of Maremmas and livestock on each are shown in Table 1A and Fig. 1. On all three properties large tracts of native vegetation remained in addition to the pasture used for livestock grazing. Maremmas were free-ranging, and could easily cross stock fences. On Riversdale, the Maremmas could obtain ad-lib dry dog food from a self-feeder in a central location; on Gillingal Station and Heatherlie, the owners visited their dogs daily to feed them.

**Table 1:** Details of the three research properties.

Property	Gillingal Station			Riversdale		Heatherlie		
***1A. Management, Losses and Tracking periods***								
***Property management***								
* Location*		Eastern Victoria		North-eastern Victoria			North-eastern Victoria	
* Topography*		Hilly, elevation 250–450 m a.s.l.		Hilly, elevation 200–800 m a.s.l.			Hilly, elevation 200–900 m a.s.l.	
* Surroundings*		State forest on all sides		Grazing properties, pine plantations, native vegetation			Grazing properties, pine plantations, native vegetation	
* Size (ha)*		800		1,214			2,428	
* Livestock*		3,000 Boer and wild-caught feral goats; cattle		1,500 Merino sheep; cattle			6,000 – 8,000 Merino and crossbred sheep; cattle	
* Number of* *Maremmas*		3; 1M, 2F*		4; 3M, 1F*			7 (2011); 3F, 4M*	
							6 (2012); 2F, 4M*	
* Stock guarded by Maremmas*		Boer goats		Merino sheep			Merino and crossbred sheep	
* Stock management*		Gates left open; stock roamed the whole property		728 ha was used for sheep. 3–4 paddocks in the area were used at one time, depending on the season			Rotational grazing. Number of paddocks in use, and the size of sheep flock in those paddocks changed frequently.	
***Losses to predators#***								
* Before Maremmas*	Up to 34 adults in one night		100 adult sheep and 100% of lambs lost yearly			150–200 adult sheep and 40% of lams lost yearly		
* Purchase of Maremmas*	1999		2006			2009		
* With Maremmas*	No adults lost, small percentage of goat kids lost yearly		No adults lost, 70% lamb survival			No adults lost, 90% lamb survival		
***GPS tracking periods***								
* Estimated age of Maremmas for the majority of time they were tracked*	8, 11, 12 yrs		4, 4, 5, 5 yrs			2, 3, 3, 4, 4, 10, 11 yrs		
* Maremmas*	June – August 2009		March – August 2010			April – September 2011		
			April – September 2011			June – December 2012		
			January – August 2012					
* Sheep*	-		March – August 2010			May – September 2011		
***1B. Kernel home range sizes***								
* * ***Maremmas***								
* 95% isopleth (ha)*	31.0±3.9		2010		286.3±81.4	2011		197.0±31.6
			2011		211.5±66.1	2012 summer		915.5±154.1
			2012 summer		148.0±55.4	2012 winter		1160.9±163.4
			2012 winter		185.2±91.3			
* 50% isopleth (ha)*	4.1±0.4		2010		20.4±4.3	2011		55.2±9.2
			2011		19.3±5.1	2012 summer		173.2±45.9
			2012 summer		14.8±4.9	2012 winter		252.1±40.6
			2012 winter		13.8±8.0			
* * ***Sheep***								
* 95% isopleth (ha)*			69.2±14.9			94.4±3.2		
* 50% isopleth (ha)*			10.8±3.1			15.9±5.7		

* M – male, F – female.

# Information on losses of livestock before and after obtaining LGDs has been sourced from records kept by the farmers themselves.

On all three properties, the main predators of livestock were wild dogs, but smaller predators were also present, including red foxes (*Vulpes vulpes)* and wedge-tailed eagles (*Aquila audax*). Predators had caused large losses of livestock on all three properties before introduction of the Maremmas; after the Maremmas started working, losses greatly decreased (Table 1A). In addition to using Maremmas for predator control, trapping, shooting and baiting of wild dogs still occurred in the vicinity of all properties, and Heatherlie was partly enclosed by an electrified wild-dog exclusion fence.

### Data collection

Movements of Maremmas were recorded using Quantum 4000 Enhanced GPS tracking collars (Telemetry Solutions, Concord, USA), which were set to take a location every 30 min, 24 hours a day. This schedule was chosen as the best trade-off between the objective to collect detailed movement data, and the estimated battery life of the collars. The majority of the GPS data was collected in autumn/winter (1 Mar –1 Sept), as the main predators in the area (wild dogs and foxes) have their breeding season in autumn, followed by birth of their young in winter. During this time predators make more incursions into livestock areas, and predation rates are higher than in spring/summer ([14], A.Bowran and M.Fraser pers. comm.). Tracking data were collected during spring/summer only on Riversdale in 2012 and Heatherlie in 2012 (Table 1A). In summer, mean temperatures range between 16.2°C at night and 30°C during the day, with a mean monthly rainfall of 46 mm. In winter, mean temperatures range between 4.9°C at night and 13.3°C during the day, with a mean monthly rainfall of 73.6 mm.

On Gilingal Station and Riversdale, all the Maremmas were collared in each tracking period (Table 1A). On these two properties, all resident Maremmas functioned as one social group. On Heatherlie, five out of seven Maremmas (two females and three males) were collared in 2011; at that time these dogs formed three distinct social units using sections the property 1–3 km apart. In 2012 one Maremma had died of old age, and all six remaining Maremmas (two females and four males) were collared. Four of these dogs (one female and three males) formed one social group that was predominantly responsible for protecting all livestock over the whole property. One female was old and mostly solitary, and one male suffered extreme social exclusion by the other individuals which severely restricted his movements. This last male was excluded from the analysis. The animals that formed part of a social unit were often found together, but social units also regularly split into sub-groups or individuals for varying lengths of time. All Maremmas were desexed, except for the male on Gillingal Station. This male had been kept sexually intact by his owner for breeding purposes, however, at the time of the research he was no longer used for breeding. Due to his old age his owner did not neuter him, since all other dogs on the property were desexed. On each of Riversdale and Heatherlie, four sheep were fitted with G2C 181 GPS tracking collars (Sirtrack, Hawkes Bay, New Zealand, Table 1A), also programmed to take a location every 30 min, 24 h a day. Maremmas were tracked for an average of 115±11 days (range: 9–174 days) before the collar failed or the battery ran out. On Riversdale the sheep were tracked for 114 days, on Heatherlie for 135 days, at which point the collars were removed because the dog tracking had ceased. The tracking data collected in this study are stored in the Movebank Data Repository [15].

### Spatial analysis

Only locations from the GPS collars with an HDOP (Horizontal Dilution of Precision) value of <4 were included in the analysis. This HDOP value was chosen based on a pilot study. In this study, all GPS collars were kept stationary on the lower branches of an apple tree for four days, taking hourly locations. Based on these data, the HDOP value was selected that offered the best balance between filtering out inaccurate locations and data retention. A mean of 1.5% of each dataset was rejected due to an HDOP value that was too high, and the mean HDOP for the remaining locations in each dataset was 1.3. Due to the large size of the datasets that were collected, the loss of a small percentage of accurate locations as a result of applying this filter was not considered a problem [16].

#### Kernel home ranges and movement analysis

Fixed kernel density distributions [17] were calculated for each dog and sheep for each tracking period to determine home range sizes. Autocorrelation does not affect the accuracy of kernel home range estimates as long as the time interval between successive locations remains relatively constant, and the number of locations is large [18]; accuracy often improves with a shorter time interval despite increased autocorrelation [18]. Therefore, we did not consider autocorrelation to be a problem for our analysis [18]–[20]. We used an *ad hoc* smoothing parameter designed to prevent under- or over-smoothing, which involved choosing the smallest increment of the reference bandwidth (Href) that results in a 95% home range polygon that was as contiguous as possible, that is, containing no, or the minimum number of, separate activity areas [21]–[23]. For each dog group, the amount of overlap between the ranges of individuals was calculated. First, this was done on a pair wise basis; for all possible combinations of dog pairs within one group the overlap of home range was determined for the 50% and 95% kernel isopleth range. For each individual, the mean overlap with any of its group mates was calculated. Second, the overlap of home ranges of all Maremmas in the group was determined. Overlap of home ranges could not be calculated for sheep, as the sample size of collared individuals in one paddock was too low in most cases.

To investigate whether behaviour changed depending on location within the home range, kernel isopleths (i.e. probability contours) were calculated for each 10% increase in kernel density. Mean movement speed (m/hour) was calculated for each individual within each isopleth. In addition, path tortuosity was used to investigate how convoluted or straight the movement path was, depending on the location in the home range. For each dataset, the total movement path was divided into separate paths in each of three areas in the home range: within the 50% isopleth, between the 50% and 90% isopleths and outside of the 90% isopleth (figure 1). A path started when the animal crossed into an isopleth area, and ended when it crossed into the next area. Tortuosity was calculated as L/R^2^, where L is the path length, and R is the net displacement (the distance in a straight line between start point and end point). We choose R^2^ as opposed to R, as R^2^ commonly increases linearly with path length [24]. Tortuosity may be scale-dependent [24], so we measured tortuosity for path segments in four lengths: 0.1, 0.25, 0.5 and 1 km. For each scale of analysis, we partitioned each separate path into sequential segments of length (L). This led to a large number of path segments, and therefore tortuosity measurements, within each isopleth area for each individual at each scale of analysis. In order to get a representative measurement of tortuosity for each isopleth area at each scale of analysis for each individual, the median of the tortuosity values of all path segments in the corresponding dataset was calculated. The mean could not be used due to the presence of a small number of outliers with extremely high values for tortuosity in most datasets.

#### Association with livestock

The presence of a Maremma in a paddock containing livestock does not necessarily indicate that the dog is closely associating with the livestock at that time. However, it does indicate that the Maremma is within a certain distance of its stock, and, in addition, that the dog is choosing to utilise the same area as the livestock. Therefore, the percentage of locations of the Maremmas that fell within sheep or goat paddocks was used as a measure to determine the association between the dogs and livestock.

To test for differences in behaviour with and without livestock, mean movement speed (m/hour), and path tortuosity were calculated for each of the two categories for each Maremma. Tortuosity was calculated as for the kernel isopleth areas, with, in this case, the total movement path of each dataset divided into separate paths in two areas; in sheep paddocks and outside sheep paddocks. A path started and ended when the animal crossed from one area into the other. If a Maremma spent more than 2 hours in close proximity to the owners' homestead, that part of the movement path was excluded from the analysis, as the motivation for those particular movements was presumably unrelated to animal-guarding activities. The separate movement paths were analysed at the four length scales described above for the isopleth areas, and median tortuosity values of the segments were calculated at each scale of analysis for each area (in or out of sheep paddocks) for each individual. Maremmas that were located in livestock paddocks 100% of the time were excluded from this analysis.

To determine which time of the day Maremmas were most likely to leave their livestock, for each category (with and without livestock), the percentage of the total number of locations within that category was calculated per hour in a 24 hour period. Then for each hour, the percentage with livestock was subtracted from the percentage without livestock.

#### Sequential use of territory

Minimum convex polygons (MCPs) were calculated for the area used each day (from midnight to midnight) per individual per tracking period, following the method of Demma and Mech [25]. MCPs were considered the best method for this analysis, as the objective was to determine the extent of the daily area of use, without regard to the density distribution of the locations. The main MCP biases were minimised by using only locations with an HDOP of <4.0, using an adequate number of GPS locations for the calculation of each MCP (48 locations per 24 hour period), and the lack of significant geographic barriers to movement [26]. The wild dog exclusion fence on Heatherlie did not influence the calculations, as no GPS locations ever occurred outside of fence, and lines from the MCPs never crossed the fence.

The average percentage of overlap of the MCP of consecutive days was determined per individual. Sequential use was defined as a daily overlap of <50%. To test for recurring patterns of re-use of specific areas of the home range, we determined the percentage of overlap during periods of 20 consecutive days for all datasets. To do this, we first determined the percentage of overlap between the first day in the series and each of the 19 consecutive days. We then took the second day as the initial day and repeated the process with the next 19 consecutive days, etc. Data were averaged across day number per individual.

#### 24-hour activity pattern

The mean movement speed per hour was calculated for each individual Maremma and sheep, and compared across the 24 hour daily cycle.

All spatial analyses were done using ArcGis 10 [27] and custom written code in R [28].

### Statistical analysis

Averages are given ± SE. Repeated measures ANOVA was used to test for statistical differences in variables for categories of analysis within each species, for the effect of age of the dog, differences between the different properties, and to test for differences between species. To test for differences between species, data was only used from the time period in which both species were collared simultaneously. The main part of the analysis focuses on data from autumn/winter only. On Riversdale and Heatherlie in 2012, variables were calculated for each individual dog for summer and winter separately, and tested for significant differences using repeated measures ANOVA. All tests used were two sided with a 95% confidence level. All statistical analysis were done using R Statistical Software [28].

## Results

### Home ranges

**Figure 2:**
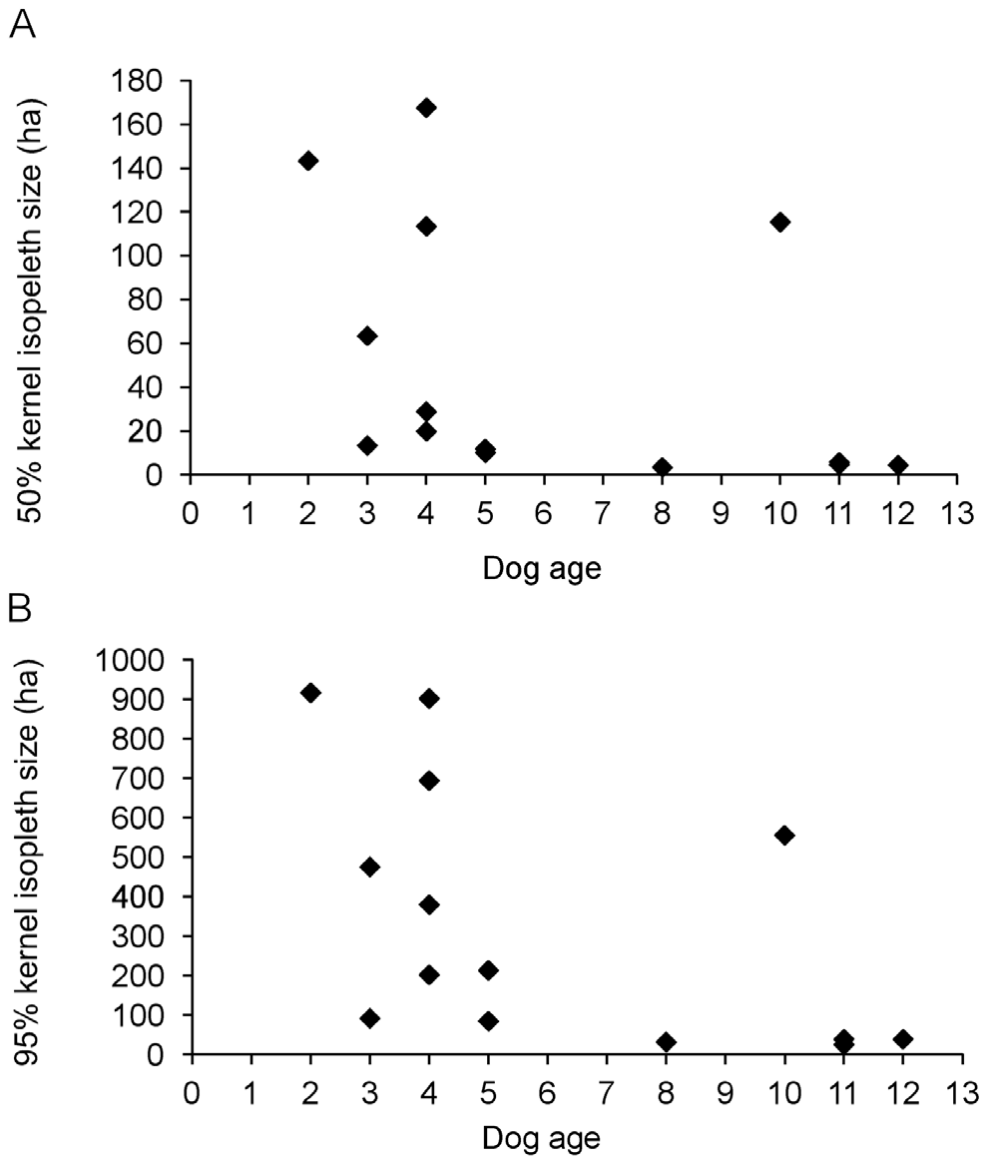
Relationship between age of the Maremma and size of the kernel isopleth home range (14 Maremmas in total). A. 50% kernel isopleth core area, and B. 95% kernel isopleth range. Each point represents the mean size of the range of an individual dog.

Kernel home range sizes at the 95% and 50% isopleths are shown in Table 1B. Home range sizes of Maremmas varied significantly with dog age (F_1, 10_ = 5.57, P<0.05); older dogs had smaller ranges (Fig. 2). As a result, the Maremmas on Gillingal Station had the smallest ranges, followed by Riversdale and then Heatherlie (Fig. 1, Table 1B), but after accounting for age, the difference between the properties was not significant (F_2,10_ = 2.81, P = 0.10). For sheep, no significant size differences were found between the home ranges on Riversdale and Heatherlie (F_1,6_ = 2.86, P = 0.14). On Gillingal Station the Maremmas' home ranges covered approximately a quarter of the property, focussing on the area that was mainly used by the purebred Boer goats (personal observation). The 50% core of the Maremmas' ranges centred on the areas where these goats camped at night. During all tracking periods on Riversdale, and the 2012 tracking period on Heatherlie, the Maremmas' entire home range (100% kernel isopleth) encompassed all the sheep-grazing paddocks, except for one paddock on Heatherlie, and extended up to two kilometres beyond them. On Riversdale, this range included sheep paddocks of neighbouring properties. The 50% core of the dog's range centred on the self-feeder on Riversdale, and encompassed parts of the main paddocks that contained sheep during the tracking period. On Heatherlie, the 50% core of the home range centred on the main parts of the main sheep grazing paddocks. In 2011 on Heatherlie, the home ranges were smaller than in 2012, due to the separation of the three dog groups in 2011. Entire home ranges (100% kernel isopleth) still encompassed all sheep paddocks on the side of the property on which the dogs were working, and extended up to one km beyond them. The 50% core of the dogs' ranges centred on the area used by the sheep to camp at night. On both Riversdale and Heatherlie, Maremmas reduced visits to paddocks when sheep were removed, and increased visits once they were present again. Maremma home ranges were significantly larger than those of the sheep they were guarding (F_1, 14_ = 9.17, P<0.01) (Table 1B). Summer and winter ranges on Riversdale and Heatherlie in 2012 were not significantly different from each other (F_1, 17_ = 1.40, P = 0.25).

The overlap of home ranges of any two Maremmas belonging to the same social group was 76.2%±3.7% for the 50% isopleth range, and 61.9%±3.4% for the 95% isopleth range (F_1, 13_ = 8.07, P<0.05). Overlap of ranges of all Maremmas within one social group was 38.4%±6.8% for the 50% isopleth range, and 54.8%±6.6% for the 95% isopleth range (F_1, 13_ = 9.92, P<0.01). The amount of overlap between pair wise home range comparisons was not significantly influenced by dog age (F_1, 11_ = 1.30, P = 0.30), however, with increasing dog age the amount of overlap between ranges of all Maremmas within a social group increased (F_1, 11_ = 7.15, P<0.05). Property did not significantly influence overlap of either pair wise home range comparisons (F_1, 11_ = 0.52, P = 0.49), or of the whole group (F_1, 11_ = 0.03, P = 0.85). There were no significant differences in amount of overlap of pair wise home ranges between summer and winter on Riversdale and Heatherlie 2012 (F_1, 15_ = 2.87, P = 0.11), although there was a trend for the overlap of ranges of the whole group to be larger in winter (F_1, 15_ = 3.43, P = 0.08).

### Activity relative to home range location

**Figure 3:**
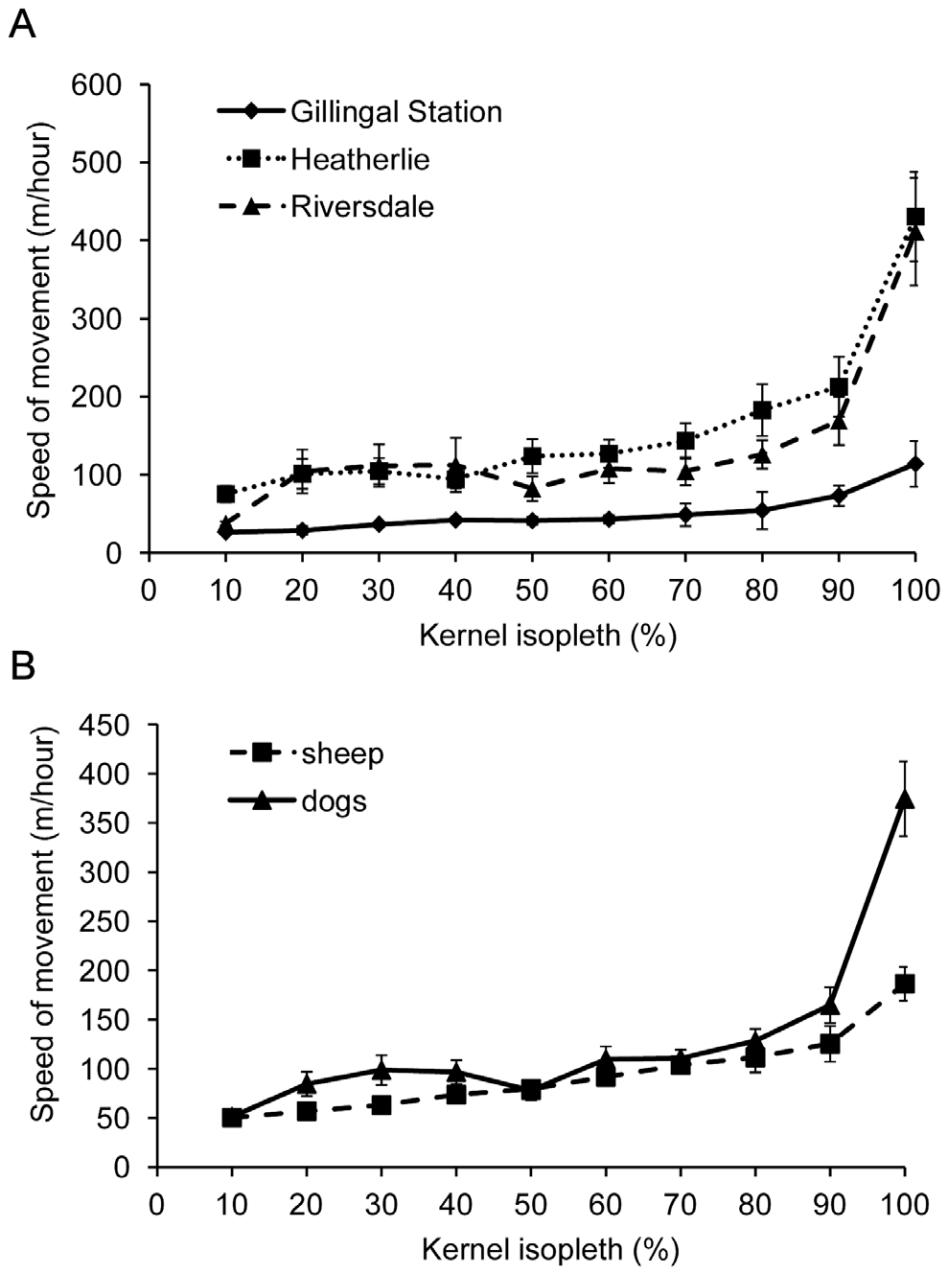
Mean speed of movement. (A) Relative to location in the kernel isopleths of Maremmas on the three properties (14 Maremmas in total). Movement speed increased significantly towards the edge of the home range (F_9, 117_ = 46.2, P<0.01), the difference between the three properties is not significant. (B) Relative to location in the kernel isopleth of Maremmas compared to sheep when they were collared at the same time (9 Maremmas and 8 sheep). Maremmas had higher speed of movement than sheep, and a greater increase in movement speed towards the edge of their home range (F_1, 10_ = 6.07, P<0.05).

Movement speed increased significantly with increasing distance from the centre of the home range for both Maremmas (F_9, 117_ = 46.2, P<0.01), and sheep (F_9, 63_ = 19.16, P<0.01). For Maremmas, overall movement speed decreased significantly with increasing age of the dog (F_1, 10_ = 9.43, P<0.05). This led to Maremmas on Gillingal Station having the lowest movement speed, followed by Riversdale and Heatherlie (Fig. 3), however, after accounting for the effect of age these differences between properties were not significant (F_2,10_ = 1.53, P = 0.26). No differences in movement speed for sheep were found between Riversdale and Heatherlie (F_1,6_ = 3.82, P = 0.1). Maremmas had higher speed of movement than sheep, and a greater increase in movement speed towards the edge of their home range (F_1, 10_ = 6.07, P<0.05) (Fig. 3). Maremmas' movement speed was on average 24.4±5.0 m/h higher in winter than in summer in all isopleth areas on both properties (F_1, 79_ = 14.77, P<0.01).

**Figure 4:**
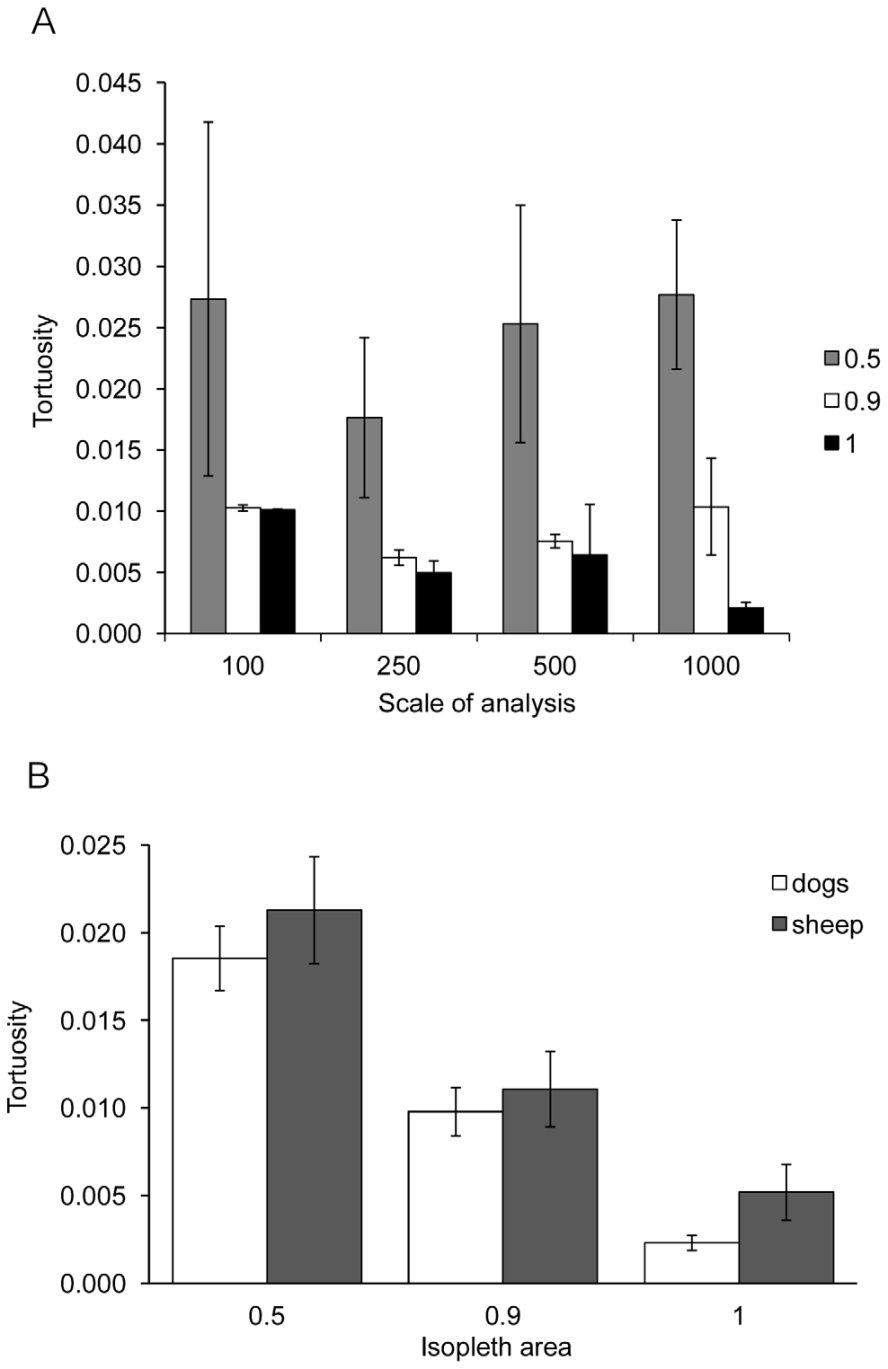
Mean median tortuosity values. A. Maremmas on the three properties within each kernel isopleth area for all scales of analysis (14 Maremmas in total). Tortuosity values decreased significantly towards the edge of the home range at all scales of analysis (0.1 km: F_2, 26_ = 4.67, P<0.05, 0.25 km: F_2, 26_ = 16.58, P<0.01, 0.5 km: F_2, 26_ = 16.17, P<0.01, 1 km: F_2, 18_ = 16.44, P<0.01). B. Maremmas compared to sheep when they were collared at the same time for the 1km scale of analysis (9 Maremmas and 8 sheep). The other scales of analysis showed a similar trend, the differences between sheep and Maremmas were not significant. Median values of tortuosity for individual dogs within each kernel isopleth area were only included in the overall calculation if the overall number of line segments used to calculate that median was equal to or greater than 10.

For Maremmas, tortuosity values decreased significantly towards the edge of the home range at all scales of analysis (0.1 km: F_2, 26_ = 4.67, P<0.05, 0.25 km: F_2, 26_ = 16.58, P<0.01, 0.5 km: F_2, 26_ = 16.17, P<0.01, 1 km: F_2, 18_ = 16.44, P<0.01) (Fig. 4). Tortuosity values in the 90%–100% and the 50%–90% kernel isopleth areas were lower than in the 50% kernel isopleth area, corresponding to straighter movement paths. In most cases the tortuosity value in the 90%–100% kernel isopleth area was also lower than in the 50%–90% area. For sheep this decrease was also found, but was significant only at three scales of analysis (0.1 km: F_2, 14_ = 2.82, P = 0.09, 0.25 km: F_2, 14_ = 4.37, P<0.05, 0.5 km: F_2, 14_ = 5.48, P<0.05, 1 km: F_2, 12_ = 8.42, P<0.01). Tortuosity values increased significantly with age for Maremmas at three scales of analysis (0.1 km: F_1, 10_ = 30.02, P<0.01, 0.25 km: F_1,10_ = 33.09, P<0.01, 0.5 km: F_1, 10_ = 37.20, P<0.01). At the 1 km scale of analysis there was no effect of age (F_1, 7_ = 1.92, P = 0.21), but 4 of the older dogs (>8 years) could not be included in this analysis as their movement paths within each isopleth area were never long enough to calculate a tortuosity value. After accounting for the effect of age, there were significant differences between properties in tortuosity values (0.1 km: F_2, 10_ = 22.82, P<0.01, 0.25 km: F_2, 10_ = 8.40, P<0.01, 0.5 km: F_2, 10_ = 5.21, P<0.05, 1 km: F_2, 7_ = 1.92, P = 0.21); the Maremmas on Gillingal Station had the highest values followed by Riversdale and then Heatherlie. There were no significant differences between Riversdale and Heatherlie in tortuosity values for sheep (0.1 km: F_1, 6_ = 0.49, P = 0.51, 0.25 km: F_1, 6_ = 0.23, P = 0.65, 0.5 km: F_1, 6_ = 0.31, P = 0.60, 1 km: F_1, 6_ = 0.41, P = 0.55). Tortuosity for sheep was higher than for Maremmas in all kernel isopleth areas at all scales of analyses (Fig. 4), but these differences were not significant (0.1 km: F_1, 14_ = 2.18, P = 0.16, 0.25 km: F_1, 14_ = 1.26, P = 0.28, 0.5 km: F_1, 14_ = 0.15, P = 0.70, 1 km: F_1, 11_ = 3.05, P = 0.11). No significant differences were found in tortuosity values for Maremmas between summer and winter on Riversdale and Heatherlie in 2012 (0.1 km: F_1, 23_ = 0.45, P = 0.45, 0.25 km: F_1, 23_ = 0.12, P = 0.74, 0.5 km: F_1, 20_ = 0.58, P = 0.46, 1 km: F_1, 20_ = 0.11, P = 0.75).

### Association with livestock

Maremmas spent significantly more time in than outside of livestock paddocks (F_1,13_ = 288.2, P<0.01), spending an average of 91.3%±2.4% of their time in livestock areas. Dog age did not significantly influence the time spent with livestock (F_1,10_ = 0.01, P = 0.92). On Gillingal station they spent 100% of their time in livestock areas, on Riversdale this was 85.6%±4.8% and on Heatherlie this was 90.9%±2.5%; these differences were not significant (F_2,10_ = 1.02, P = 0.40). There were no significant difference in the proportion of time spent with sheep between summer and winter for Riversdale and Heatherlie in 2012 (F_1,17_ = 0, P = 1).

Outside sheep paddocks, Maremmas travelled faster than inside sheep areas: 300.1 m/hour ±42.2 m/hour vs. 192.5 m/hour ±18.7 m/hour (F_1, 9_ = 13.57, P<0.01). There was a trend for overall movement speed to decrease with Maremma age (F_1, 6_ = 4.98, P = 0.06), and Maremmas on Heatherlie travelled faster than the Maremmas on Riversdale (F_2, 6_ = 9.81, P<0.05). Speed of movement in sheep paddocks was not significantly different between summer and winter for Riversdale 2012 and Heatherlie 2012 (F_1,6_ = 0.50, P = 0.50). Speed of movement outside sheep paddocks was on average 61.5±28.4 m/h higher in winter than in summer (F_1, 6_ = 9.27, P<0.05).

**Figure 5:**
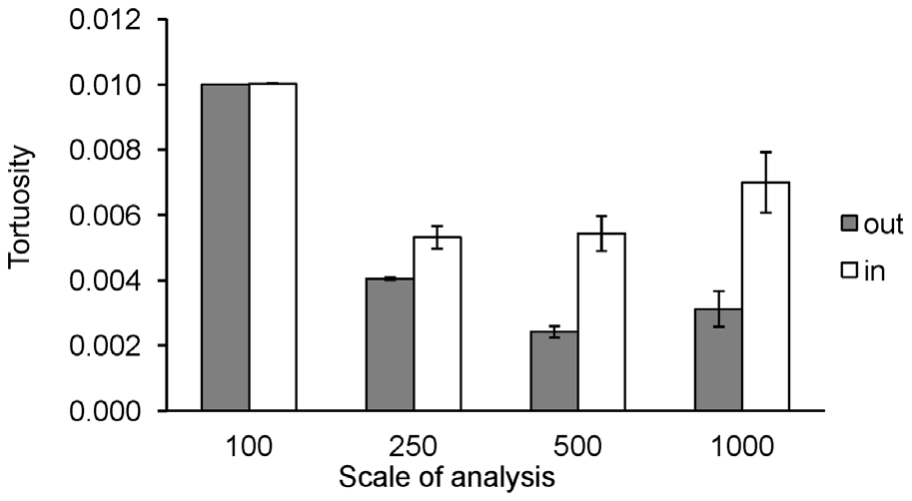
Median tortuosity values of movement paths of Maremmas inside and outside of livestock areas at four different scales of analysis (10 Maremmas in total). Inside livestock areas tortuosity values were significantly higher for all four scales of analysis (0.1 km: F_1, 9_ = 11.25, P<0.01; 0.25 km: F_1, 9_ = 14.08, P<0.01; 0.5 km: F_1, 9_ = 40.71, P<0.01 and 1 km: F_1, 9_ = 31.96, P<0.01). Median values of tortuosity for individual dogs within each livestock area were only included in the overall calculation if the number of line segments that was used to calculate that median was equal to or greater than 10.

Outside sheep paddocks, path tortuosity values for Maremmas were lower than inside sheep paddocks, indicating that movement paths were straighter (Fig. 5). This difference was significant for all four scales of analysis (0.1 km: F_1, 9_ = 11.25, P<0.01; 0.25 km: F_1, 9_ = 14.08, P<0.01; 0.5 km: F_1, 9_ = 40.71, P<0.01 and 1 km: F_1, 9_ = 31.96, P<0.01). Tortuosity values were significantly higher with increasing age of the dog at two scales of analysis (0.1 km: F_1, 7_ = 12.31, P<0.01; 0.25 km: F_1, 7_ = 5.29, P = 0.05; 0.5 km: F_1, 7_ = 2.11, P = 0.19; 1 km: F_1, 7_ = 3.02, P = 0.13). After accounting for dog age, tortuosity values in and out of sheep paddocks did not differ significantly between properties (0.1 km: F_1, 7_ = 0.01, P = 0.95; 0.25 km: F_1, 7_ = 0.21, P = 0.66; 0.5 km: F_1, 7_ = 0.01, P = 0.92; 1 km: F_1, 7_ = 0.09, P = 0.77), and were not significantly different between summer and winter for Riversdale 2012 and Heatherlie 2012 (0.1 km: F_1, 15_ = 0.79, P = 0.39; 0.25 km: F_1, 13_ = 0.35, P = 0.57; 0.5 km: F_1, 13_ = 0.48, P = 0.50; 1 km: F_1, 13_ = 0.38, P = 0.55).

**Figure 6:**
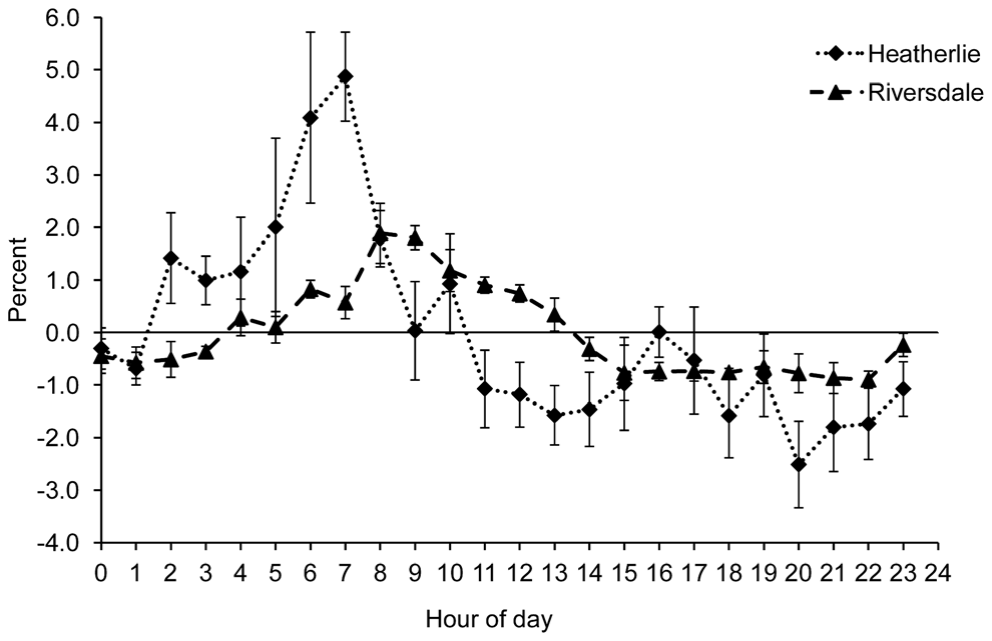
The time of the day at which Maremmas are more likely to leave their livestock (10 Maremmas in total). When the graph is positive, it indicates the times of the day that Maremmas are more likely to leave stock, when the graph is negative, it indicates the times of the day that Maremmas are less likely to leave stock. This number is calculated as follows: the percentage of the total number of locations for each category (with and without livestock) that fell within that hour of the day was calculated for each dog. Then for each hour, the percentage with livestock was subtracted from the percentage without livestock.

Maremmas were more likely to leave livestock in the early hours of the day (Fig. 6). There was no significant difference in this pattern between dogs of different ages (F_1, 7_ = 1.30, P = 0.29), between properties (F_1, 7_ = 0.04, P = 0.86), nor between summer and winter for Riversdale and Heatherlie in 2012 (F_1, 167_ = 0, P = 1).

### Sequential use of territory

For Maremmas, the average daily percentage overlap of the MCP of two consecutive days was 45.8%±2.4%. The amount of overlap in MCPs of consecutive days increased with the age of the dog (F_2, 10_ = 23.40, P<0.01). After accounting for age of the dogs, there was a trend for the Maremmas on the different properties to have different MCP overlap values; on Gillingal Station daily overlap was 57.0%±4.2%, on Riversdale it was 38.2%±1.3%, and on Heatherlie it was 46.0%±2.4% (F_2, 10_ = 3.67, P = 0.06). Average daily percentage overlap of the MCP did not significantly differ between summer and winter for Riversdale and Heatherlie in 2012 (F_1,7_ = 0.82, P = 0.40). For sheep the average was significantly higher than for dogs: 54.8%±2.1% (F_1, 18_ = 10.29, P<0.01). The sheep on Riversdale had on average 11.0% more daily overlap of MCP's than the sheep on Heatherlie (F_1, 6_ = 113.1, P<0.01).

**Figure 7:**
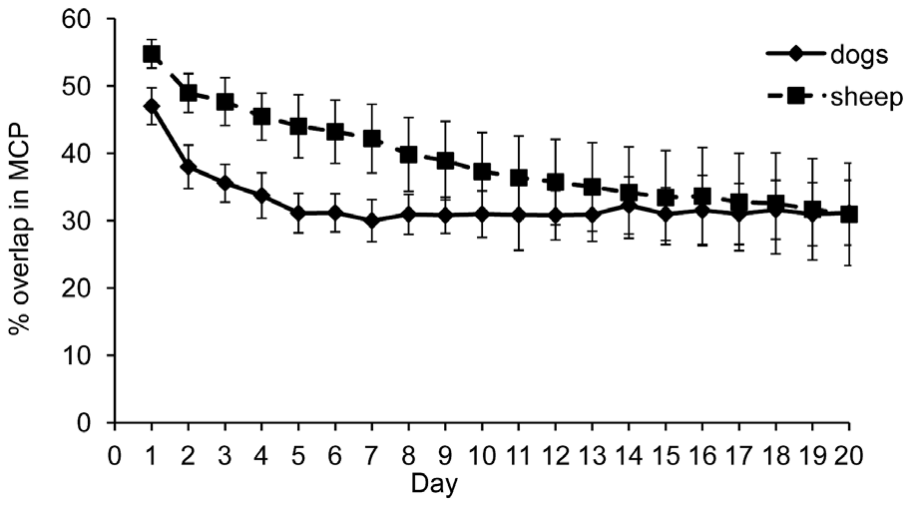
The percentage of overlap in MCP's in consecutive 20-day periods for Maremmas compared to sheep, the difference between the two species is not significant (9 Maremmas and 8 sheep). Both species were collared during the same time period. First the overlap between the first day of the series and each of the 19 consecutive days was calculated, after which the process was repeated by using the second day as the initial day, and calculating the overlap between that day and each of 19 consecutive days.

No consistent pattern of re-use of particular parts of the home range was found for either Maremmas or sheep over 20 day periods (Fig. 7). For both species the percentage of overlap between MCPs of each initial day with each of the following 19 days decreased with increasing number of days (Maremmas: F_19, 228_ = 9.49, P<0.01, sheep: F_19, 113_ = 14.57, P<0.01) (Fig. 7). For Maremmas, older dogs had significantly higher percentages of overlap in MCPs of each of the 19 consecutive days than younger dogs (F_1, 9_ = 6.22, P<0.05). After accounting for age, there were no significant differences between properties (F_2, 9_ = 1.48, P = 0.28). The sheep on Riversdale on average had 35.8%±1.76% more overlap in the MCPs of each of the 19 consecutive days than the sheep on Heatherlie (F_1, 6_ = 196.4, P<0.01), due to the highly regular paddock rotation schedule on Heatherlie. There were no significant differences in the pattern of overlap of MCPs between Maremmas and sheep (F_1,14_ = 0.54, P = 0.48). However, if the sheep on Heatherlie are excluded, sheep have on average 20.0%±0.5% more overlap in MCPs in each of the 19 consecutive days than Maremmas (F_1, 10_ = 14.20, P<0.01). For Maremmas, the decrease in overlap of MCPs was also seen in summer and winter on Riversdale and Heatherlie in 2012, but there was a trend for summer to be different from winter (F_1, 139_ = 3.64, P = 0.06). This was mainly caused by the percentage overlap between day one and each consecutive day being higher in the last three days in summer compared to winter.

### 24-hour activity patterns

**Figure 8:**
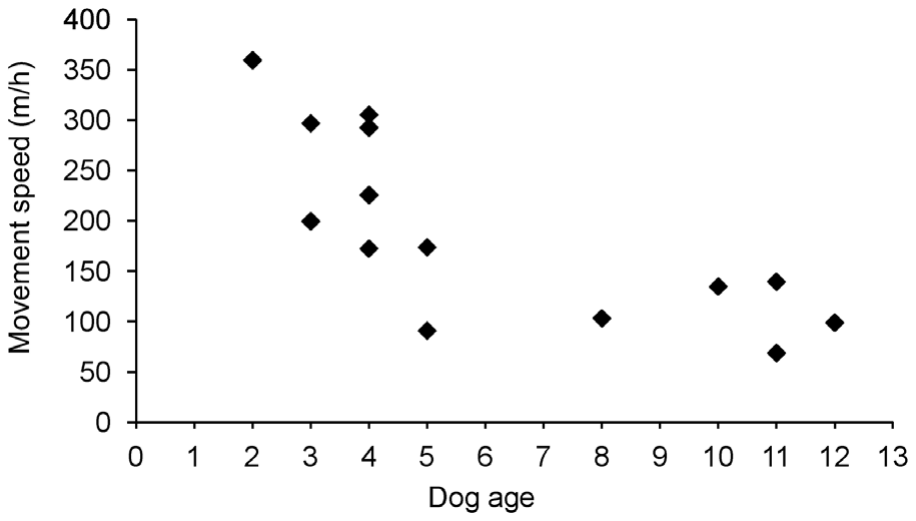
Relationship between age of the Maremma and movement speed (m/h). Each point represents the mean movement speed of an individual dog (14 Maremmas in total).

Average hourly movement speed of Maremmas significantly decreased with increasing dog age (F_1, 10_ = 28.26, P<0.01) (Fig. 8). There were also significant differences between the properties (F_2, 10_ = 5.47, P<0.05), being lowest on Gillingal Station (91.5±11.4 m/hour), followed by Riversdale (165.4±27.9 m/hour) and Heatherlie (245.9±33.7 m/hour). In winter, Maremmas travelled on average 35.3 m/h more than in summer (F_1,7_ = 10.84, P<0.05). Sheep on Riversdale had a significantly higher average movement speed than sheep on Heatherlie (F_1, 6_ = 7.20, P<0.05); 150.6±8.1 m/hour on Riversdale versus 115.7±10.7 m/hour on Heatherlie. Maremmas had a significantly higher average movement speed than the sheep they were guarding (F_1, 14_ = 7.93, P<0.05).

**Figure 9:**
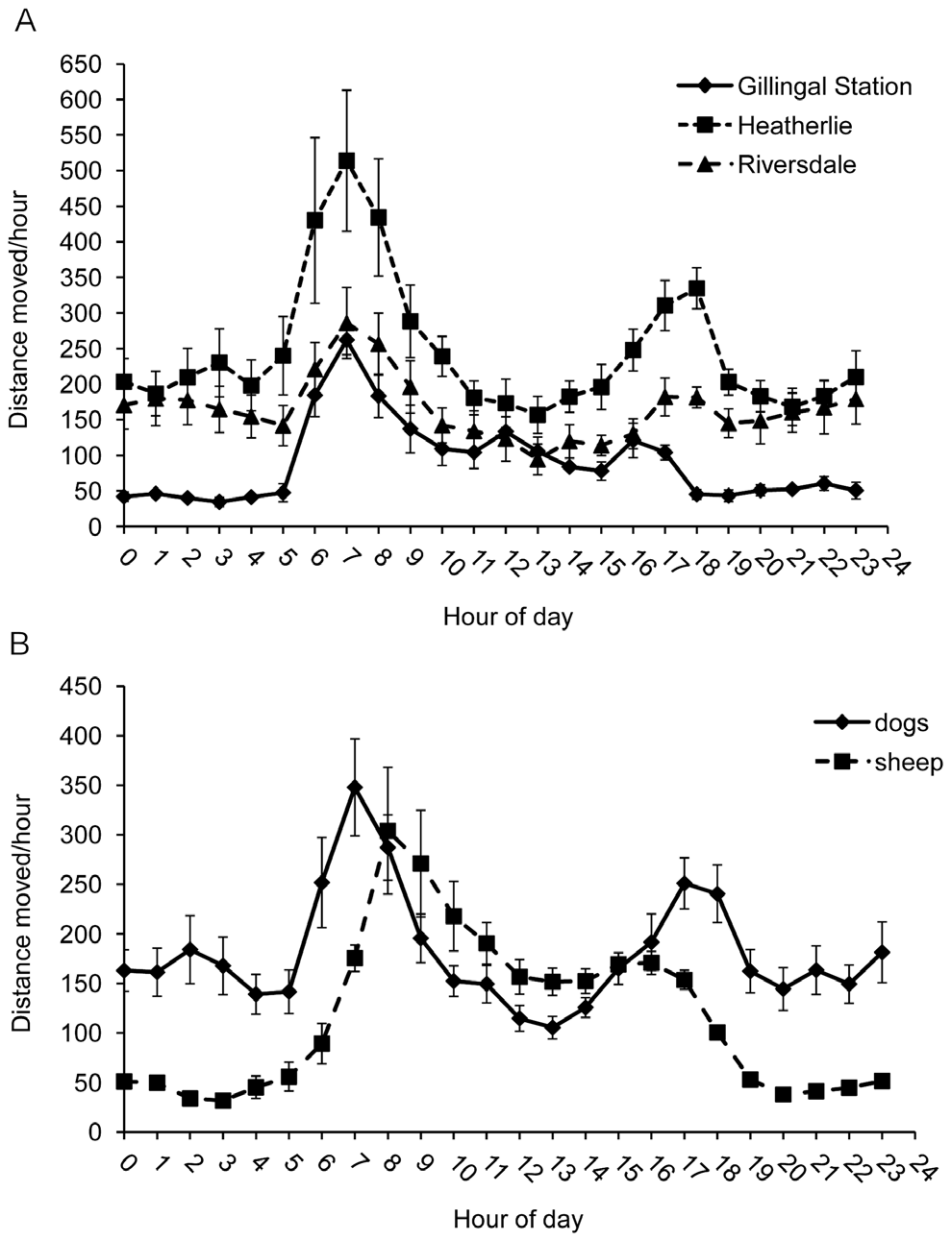
Average 24-hour activity patterns. (A) Maremmas on the three research properties (14 Maremmas in total). The overall level of activity was significantly different between the three properties F_2, 10_ = 5.47, P<0.05. (B) Maremmas compared to sheep when both were collared during the same time period (9 Maremmas and 8 sheep). The activity pattern was significantly different between the two species (F_1, 14_ = 7.49, P<0.05). The Y-axis represents the average distance moved per hour in a 24 hour period.

Figure 9 shows the 24-hour activity pattern of both Maremmas and sheep. Both species had a distinct early morning peak in activity after sunrise and a late afternoon peak in activity before sunset. The activity pattern was significantly different between the two species (F_1, 14_ = 7.49, P<0.05). The morning activity peak began earlier for Maremmas than for sheep, and activity peaked later in the afternoon. In addition, Maremmas maintained a higher level of activity at night than sheep. The pattern of activity in a 24 hour period was very similar between different aged dogs, and between properties for the Maremmas, but with increasing age the mean activity per hour over the whole 24-hour period for dogs decreased (F_1, 10_ = 28.26, P<0.01), and there were differences between the different properties (F_2, 10_ = 5.47, P<0.05). The Maremmas on Gillingal station were least active, followed by Riversdale and then Heatherlie (Fig. 9). There was a significant difference in activity pattern between sheep on Riversdale and Heatherlie (F_1, 6_ = 7.20, P<0.05), mainly due to a higher morning activity peak of the sheep on Heatherlie.

The 24-hour activity pattern was significantly different between summer and winter for the dogs on Riversdale and Heatherlie in 2012 (F_1,191_ = 43.28, P<0.05). The timing of the morning and afternoon activity peaks shifted as sunrise and sunset times changed, and in addition, in winter the dogs had a slightly overall higher activity level than in summer.

## Discussion

Unsupervised free-ranging Maremmas spent the majority of their time in livestock areas, apparently choosing to remain in proximity to their livestock most of the time. However, movements away from livestock did occur, mainly at night. These movements were generally characterised by high-speed travel on relatively straight paths. These high-speed movements were related to the finding that movements become faster towards the edge of the range boundary, because the Maremmas' ranges encompassed the livestock areas, and therefore leaving the livestock paddocks entailed moving towards the range edge. Travel between livestock paddocks, and between livestock paddocks and the owners' homestead, accounted for a number of straight high-speed movements. In other cases, fast movements away from livestock could represent responses to predator incursions or perceived predator threats, or simply exploratory behaviour. Alternatively, the movements could be related to patrolling territorial boundaries. No published research has as yet provided clear evidence that LGDs are territorial, and might use territorial exclusion of predators to protect livestock. However, a number of studies have recorded the use of territorial signalling by LGDs, such as scent-marking, regular barking and boundary patrolling [29]–[33].

Movements of sheep also became faster and less tortuous towards range edges, but less so than for Maremmas. Overall, sheep movements were always more tortuous than dogs'. This probably reflects different motivations for movement between the two species. In the case of the sheep, water was plentiful on both properties, and movements were mainly governed by grazing and resting patterns. In the case of the dogs, food was provided by their owners and water was easily obtained. The movements in the core of their range in livestock areas probably reflect activities such as resting, play and following livestock. Movements towards the edge of their range could be motivated by a number of factors (as explained above).

In pair wise comparisons, Maremmas belonging to the same social group had a relatively high degree of overlap of 50% and 95% isopleth ranges, indicating that any two dogs within the same group share a large portion of their home range. The amount of overlap of the ranges of all members of the same group was lower, especially in the 50% isopleth. This indicates that members of the same social group, while sharing a large part of their home range with the other dogs in the group, do all occupy slightly different areas, especially at the core of their range. Therefore, the group as a whole occupies a larger area than each individual dog on their own, which could potentially increase their effectiveness for livestock protection. The relatively low percentage of overlap between areas used by the same dog on consecutive days points to sequential use of the home range by the Maremmas. In sheep, the amount of overlap between areas used on consecutive days was higher than for dogs, but only slightly. In sheep this behaviour is probably caused by their grazing patterns. Sequential use of the home range would probably optimise foraging for sheep, as the quality of areas recently grazed has declined and new areas offer a better alternative [34]. If the Maremmas consistently move with their sheep, this could explain a large part of the sequential use of the range by the dogs. In addition, for the dogs, traversing a different area of the range each day probably facilitates maintaining a presence throughout the whole range.

The activity patterns of Maremmas show that they are most active in the early morning and late afternoon, and maintain a relatively high level of activity during the night. This activity pattern matches that of the main predators in the areas where the dogs were working [35]–[38]. Compared to the Maremmas, the peaks of sheep activity were later in the morning and earlier in the afternoon, and sheep were relatively inactive all during the night. Consistent daily sheep activity patterns have also been documented in other studies [39]–[41]. Late start of grazing and earlier bedding down seems to be related to season [41], and is probably the result of this study taking place mostly in autumn and winter.

Dog age significantly influenced the general level of activity of the Maremmas, with Maremmas displaying less activity with increasing age. Older dogs moved less per hour, and their movement paths tended to be more tortuous. This decrease in activity likely leads to smaller home ranges, and therefore the decrease in home range size with increasing age of the dog that was observed in this study. This probably means that as a LGD gets older, the size of the area in which it can effectively protect livestock decreases. One way to counter this effect would be to have older LGDs working together with younger dogs. The older LGDs can teach the younger dogs the job [11], and as the younger dogs maintain a higher level of activity and have a larger home range, they still protect the livestock over the larger area that the older LGDs used to occupy.

Other than a higher overall level of activity in winter, and small differences in the patterns of re-use of territory, no differences were found between movements of Maremmas in summer and winter. The higher activity of the Maremmas in winter could be related to the breeding season of wild dogs and foxes, which can lead to an increase in incursions in livestock areas [14]. This would require a higher level of vigilance from the Maremmas, which could lead to higher activity. Other than that, it seems that seasonality has little effect on Maremma movements, other than a slight adjustment to different sunrise/sunset times. This is probably due to two main factors. First, food was provided by their owners, so foraging was not necessary, and water was plentiful on all properties throughout the year. Second, most Maremmas were desexed, and mating and breeding related behaviours did not occur in different seasons.

Proper management of pups, and later of adult dogs, is crucial for LGDs to develop the behaviour which causes them to choose to spend the majority of their time with livestock. Pups need to be thoroughly socialised to the species they need to protect later in life; without proper bonding to livestock as a pup, a mature LGD is unlikely to voluntarily remain with livestock unsupervised for any length of time [2], [42], [43]. In a free-range system, some movements away from livestock are always likely, no matter how well-bonded the dog is to its stock. However, van Bommel & Johnson [10] found that the management style of LGDs (free-ranging vs. restricted in movements, i.e. fence trained or not) did not influence how well LGDs were able to protect their livestock. On large Australian properties where wild dogs or dingoes are the main predator, free-ranging of LGDs is often considered by livestock managers to be the most effective management strategy, as LGDs are able to provide each other with backup in case of a predator attack [10], [44]. This would not be possible if LGD movements were restricted. Territoriality could explain why LGDs can still be effective in protecting stock even though they do not spend all their time with them. If LGDs set up a territory around the livestock, and predators are deterred by those territorial boundaries, then the LGDs continuous presence with livestock is not required to keep the stock safe. On the contrary, some movement away from stock is actually necessary for the LGDs to patrol and maintain territorial boundaries. More research is needed to investigate if or to what extend LGDs work through territorial exclusion of predators.

When producers are faced with the management decision of whether to have their LGDs free-ranging or to restrict their movements, the potential size of the range that the LGDs will use should be taken into consideration, and compared to the size of the property that the dog is meant to work on. We found that 95% kernel isopleth ranges of LGDs measured up to 1161 ha. The size of the range is likely influenced by many variables, and will sometimes be smaller, but sometimes also larger. One factor that is potentially important in determining the size of the dogs' range is the combined size of the ranges of the different groups of livestock they are guarding. However, regardless of the size of the area that the livestock use, LGDs are likely to have a larger range than the stock. In this study the boundaries of the Maremmas' ranges extended up to 2 km beyond the boundaries of the ranges of the stock, and in the case of the Maremmas on Riversdale, the dogs even chose to include the sheep on a neighbouring property in their range. These factors should all be taken into consideration when deciding if a LGD can operate in a free-range system. If a property is smaller than the potential size of the range of the dogs, free-ranging might not be the best management decision, and the choice can be made for example to fence train the LGD, or to upgrade boundary fences to prevent the dog leaving the property. Roaming by dogs outside their property boundaries could lead to problems such as traffic accidents, concerned neighbours, or conflicts with local councils, as in most Australian states dogs are required by law to be contained within their owners' property if not accompanied by a human. However, if the property can accommodate the range that the LDGs will use, free-ranging can be an effective management system, allowing the LGDs to maintain a presence, and deter predators, throughout and around the livestock areas.
